# Age-Dependent Regulation of Notch Family Members in the Neuronal Stem Cell Niches of the Short-Lived Killifish *Nothobranchius furzeri*

**DOI:** 10.3389/fcell.2021.640958

**Published:** 2021-07-09

**Authors:** Sara Bagnoli, Eva Terzibasi Tozzini

**Affiliations:** ^1^Laboratory of Biology (BIO@SNS), Scuola Normale Superiore, Pisa, Italy; ^2^Department of Biology and Evolution of Marine Organisms (BEOM), Stazione Zoologica Anton Dohrn, Naples, Italy

**Keywords:** notch pathway, ageing, killifish, adult neurogenesis, *in situ* hybridization, RNA sequencing

## Abstract

**Background:** The annual killifish *Nothobranchius furzeri* is a new experimental model organism in biology, since it represents the vertebrate species with the shortest captive life span and also shows the fastest maturation and senescence recorded in the laboratory. Here, we use this model to investigate the age-dependent decay of neurogenesis in the telencephalon (brain region sharing the same embryonic origin with the mammalian adult niches), focusing on the expression of the Notch pathway genes.

**Results:** We observed that the major ligands/receptors of the pathway showed a negative correlation with age, indicating age-dependent downregulation of the Notch pathway. Moreover, expression of *notch1a* was clearly limited to active neurogenic niches and declined during aging, without changing its regional patterning. Expression of *notch3* is not visibly influenced by aging.

**Conclusion:** Both expression pattern and regulation differ between *notch1a* and *notch3*, with the former being limited to mitotically active regions and reduced by aging and the latter being present in all cells with a neurogenic potential, regardless of the level of their actual mitotic activity, and so is less influenced by age. This finally suggests a possible differential role of the two receptors in the regulation of the niche proliferative potential throughout the entire fish life.

## Introduction

In the last decade, our group has intensively studied the annual fish *Nothobranchius furzeri* as a new experimental model in biology. *N. furzeri* has gained recognition as an animal model for aging studies, and currently, it is extensively studied in an increasing number of laboratories worldwide (Kabiljo et al., 2019; Matsui et al., 2019; Montesano et al., 2019; Fumagalli et al., 2020; Hu et al., 2020; Kelmer et al., 2020; Wang et al., 2020). These fish inhabit ephemeral pools in the semiarid bushveld of Southern Mozambique characterized by scarce and erratic precipitations and have adapted to the seasonal drying of their environment by producing desiccation-resistant eggs that can remain dormant in the dry mud for 1 and maybe more years by entering into a diapause state (Genade et al., 2005; Cellerino et al., 2016; Kim et al., 2016). Due to very short duration of the rainy season, the natural life span of these animals is limited to a maximum of a few months (Terzibasi Tozzini et al., 2013; Dorn et al., 2014; Vrtílek et al., 2018). They represent the vertebrate species with the shortest captive life span and also the fastest maturation (Genade et al., 2005; Blazek et al., 2013). In addition, they express a series of conserved aging markers and are amenable to genetic manipulations, making them an attractive model system for aging research (Valenzano et al., 2006, 2011; Terzibasi et al., 2007; Hartmann et al., 2009, 2011; Reichwald et al., 2009; Di Cicco et al., 2011; Hartmann and Englert, 2012; Tozzini et al., 2012; Allard et al., 2013), as reviewed in Cellerino et al. (2016) and Kim et al. (2016). The recent sequencing of the *N. furzeri* genome (Benayoun et al., 2015; Reichwald et al., 2015; Valenzano et al., 2015) and the establishment of transgenic techniques for this species (Valenzano et al., 2011; Hartmann and Englert, 2012; Benayoun et al., 2015; Harel et al., 2015; Ripa et al., 2016; Dolfi et al., 2019) made *N. furzeri* a new versatile model organism to investigate the genetic basis of aging (Platzer and Englert, 2016; Cui et al., 2020). A striking characteristic of killifish development is the presence of diapause, a period when embryonic development is suspended at specific stages (Furness et al., 2015; Hand et al., 2016), such as gastrulation (diapause 1), mid-somitogenesis (diapause 2), and pre-hatching (diapause 3). A surprising result that recently emerged is that the transcriptional patterns observed during aging and diapause overlap to a significant extent (Reichwald et al., 2015), providing the motivation to study development-related genes in the context of aging.

Neurogenesis is the process that generates the nervous system during embryonic development. In mammals, neurogenesis is completed during perinatal life and is restricted to specific telencephalic niches during adult life (Ming and Song, 2011; Urbán and Guillemot, 2014), but in fish brains, adult neurogenesis remains active, with stem cell niches distributed along the entire rostro-caudal extent of the ventricular surface (Zupanc and Horschke, 1995; Adolf et al., 2006; Grandel et al., 2006; Kuroyanagi et al., 2010). Adult neurogenesis in mammals is known to decrease dramatically with age (Kuhn et al., 1996; Pekcec et al., 2008; Ben Abdallah et al., 2010; Knoth et al., 2010), and although the level of adult neurogenesis remains higher compared to mammals, a decrease of this phenomenon has been observed during aging in zebrafish as well (Edelmann et al., 2013; Kaslin et al., 2013; Stankiewicz et al., 2017). We therefore studied adult neuronal stem cells (NSCs) of *N. furzeri in vivo* and demonstrated an age-dependent decay in adult neurogenesis in terms of both incorporation of nucleotide analogs and expression of specific markers (Tozzini et al., 2012). In addition, RNA sequencing (RNA-seq) experiments revealed age-dependent downregulation of neurogenesis genes during aging of *N. furzeri* brain and identified novel markers of adult NSCs (Baumgart et al., 2014).

Even though fish neurogenic niches are more widespread than the mammalian homologous structures, the telencephalic niches share the same embryonic origin with the mammalian adult niches (Adolf et al., 2006). In particular, the ventral niche is homologous to the mammalian subventricular (subpallial) zone, whereas the dorsolateral region is homologous to the pallial zone (hippocampal mammalian niche). We also analyzed the germinal zone of the optic tectum that is very active in teleosts and whose stem cells retain a neuroepithelial phenotype (Tozzini et al., 2012). This would allow us to differentiate between conserved expression patterns in the telencephalic niches from possible teleost-specific expression patterns detectable only in the optic tectum.

We concentrated our attention on the Notch pathway during aging of the NSCs. The Notch pathway is a key pathway for the control of neurogenesis and cell fate during both embryonic development (Yoon and Gaiano, 2005; Pierfelice et al., 2011; McIntosh et al., 2017; Kanungo et al., 2018) and adult neurogenesis (Suh et al., 2009; Dozawa et al., 2014; Than-Trong et al., 2018; Sueda and Kageyama, 2020). When activated by the ligands (Delta and Jagged), the Notch intracellular domain is cleaved and activates the transcription of effectors of the hairy family (*her* and *hes* genes in vertebrates) that act as repressors of gene transcription that block neurogenic genes (Yeo et al., 2007; van Tetering and Vooijs, 2011). Expression of *notch* genes is retained in adult NSCs where Notch activity can be detected by the activity of the *hes5* promoter (Lugert et al., 2010). In zebrafish, *notch* family is composed of four members: *notch1a*, *notch1b*, *notch2*, and *notch3* (Bierkamp and Campos-Ortega, 1993; Westin and Lardelli, 1997). Studies in adult zebrafish have revealed a differential activity of *notch1* and *notch3*. Inactivation of *notch3* induces NSC amplification by reactivating them from a quiescent status, promoting symmetric divisions. On the other hand, abrogation of *notch1* function induces new neuron generation, inducing a reduction of the number of activated NSCs (Alunni et al., 2013; Than-Trong et al., 2018). Effects of aging on the Notch pathway remain poorly investigated. We reanalyzed RNA-seq and proteomics data to specifically assess the age-dependent expression of the components of the Notch pathway, and we studied the expression of *notch1* and *notch3* using *in situ* hybridization (ISH).

## Materials and Methods

### Fish Breeding and Housing Conditions

All experiments were performed on group-house *N. furzeri* of the MZM-04/10 strain. The protocols of fish maintenance were carried out in accordance with all animal use practices approved by the Italian Ministry of Health (Number 96/2003a). Ethynyl deoxyuridine (EdU)-injected samples used for the experiments were derived from archived material of the animal stock as previously used by Tozzini et al. (2012).

Eggs were maintained on wet peat moss at room temperature in sealed Petri dishes. When embryos had developed, eggs were hatched by flushing the peat with tap water at 16–18°C. Embryos were scooped with a cut plastic pipette and transferred to a clean vessel. Fry were fed with newly hatched *Artemia nauplii* for the first 2 weeks and then weaned with finely chopped *Chironomus* larvae. Starting at the fourth week of life, fish were moved to 40-L tanks at a maximum density of 20 fish per tank equipped with air-driven sponge filters. The aquarium room’s temperature was set at a constant 26°C. Twice a week, the bottom of the tanks was siphoned and 50% of the water was exchanged with tempered tap water.

### *In vivo* Ethynyl Deoxyuridine Injection

To localize the neurogenic niches in the brain, fishes were treated with a single intraperitoneal injection of the modified thymidine analog EdU (5-ethynyl-2’-deoxyuridine), which is specifically incorporated in the DNA during the S-phase of proliferating cells. To do that, the Click-iT^®^ EdU Alexa Fluor^®^ 488 Imaging Kit from Invitrogen-Thermo Fisher Scientific (cat. no.: C10337) was used: fish were first shortly anesthetized by using a sedation dosage of tricaine methanesulfonate (MS-222), and 50 μl of a 10-μM EdU solution was injected intraperitoneally in the experimental fish. Finally, animals were euthanized with MS-222 4 h after injection and immediately dissected. Preparation and use of MS222 and all procedures conform to the prescription of European (Directive 2010/63/UE) and Italian laws (DL 26/04-03-2014).

### Brain Tissue Collection and Preparation

Whole brains from five young (7 weeks) and five old (25 weeks) animals (listed in Table 1) were dissected and fixed by immersion in 4% paraformaldehyde/0.1 M phosphate buffer (pH 7.4) and then cryoprotected with a two-step immersion at 20% and then 30% sucrose solution for at least 12 h each. Finally, the tissues were embedded at –20°C in Neg50 cryo-embedding medium (Thermo Scientific); series of 16-μm-thick sections were cut with a Leica cryostat and collected on Superfrost plus slides^®^ (Thermo Scientific).

**TABLE 1:** Total list of experimental animals used in the study.

Protocol	Fish species and strain	Age (weeks)	Animals number	*N* Tot
**RNA Seq** Data set 1 Baumgart et al., 2014	Species: *Nothobranchius furzeri* Strain: MZM04/10	5	5	25
		12	5	
		20	5	
		27	5	
		39	5	
**RNA Seq** Data set 2 Kelmer et al., 2020	Species: *Nothobranchius furzeri* Strain: MZM04/10	5	4	12
		12	4	
		39	4	
**Proteomic** Data set Kelmer et al., 2020	Species: *Nothobranchius furzeri* Strain: MZM04/10	5	5	15
		12	5	
		39	5	
		12	3	
		39	3	
**Histology (EdU-ISH)**	Species: *Nothobranchius furzeri* Strain: MZM04/10	5	5	10
		27	5	
				63

*EdU, ethynyl deoxyuridine; FDR, false discovery rate; ISH, *in situ* hybridization.*

### *In situ* Hybridization and Ethynyl Deoxyuridine Staining

#### *In situ* Hybridization

All ISH protocols have been performed on 16-μm-thick cryo-sections of fish brain. Slides were dried for 2 h at 37°C, washed in phosphate buffered saline (PBS) twice for 3 min, and then treated for 8 min with Proteinase K (diluted 1:80,000 starting from stocks of 20 mg/ml). After that, slides were washed in glycine (2 mg/ml in PBT solution = PBS + tween 20 0.1%) twice for 5 min to stop the reaction. Then, sections were fixed with paraformaldehyde (PFA) 4% for 20 min at room temperature and washed in PBT (three times for 3 min). Pre-hybridization was performed by covering the slides with 200 μl of hybridization buffer under parafilm coverslips (to avoid evaporation) at hybridization temperature (60°C) for 30 min inside a wet chamber. Hybridization was performed covering each slide with a solution of the specific antisense 3’DIG-labeled probe diluted in 500 μl of hybridization buffer to a final concentration of 1 μg/ml. Parafilm coverslips were used, and slides were incubated at hybridization temperature overnight. Before using them, diluted RNA probes have been denatured for 2 min at 94°C. In order to avoid drying out the slides, the whole process has been carried out in a wet chamber with PBS.

After hybridization, 2 × saline-sodium citrate (SSC) has been used to remove the coverslip. Slides were first washed in 2 × SSC, twice for 20 min, and then in 0.2 × SSC twice for 20 min, always at hybridization temperature. A final washing step was done in PBT three times for 5 min at room temperature.

For the probe revelation, slides were incubated with blocking solution for 30 min at room temperature and then with Anti-Dig-AP Fab Fragments Ab [1/2,000] in blocking solution overnight at 4°C.

Washing in PBT, three times for 5 min, and in NMNT (NaCL/trisHCl/MgCl2/Tween20 0,1%/ Tetramisole solution), three times for 5 min at room temperature, has been conducted before adding Fast Red solution (Roche Tablets; 1 in 2 ml *Tris–*HCl 0.1 M, pH = 8.2). To avoid the formation of precipitate, Fast Red tablets have been vortexed for 5 min in *Tris*–HCl and then filtered. Observation has been conducted every 20 min with a Zeiss fluorescence microscope until the signal detection. The staining has been stopped by washing well with PBS (at least three times for 5 min) at room temperature.

The young and old samples have been treated in parallel, and to be able compare the dimension of the expression domains between different ages, we stopped the colorimetric reaction in both at the same time. After ISH slides were treated for EdU immunolabeling, images were acquired using a confocal microscope (Leica TCS).

#### EdU Staining

After the ISH procedure, slides were processed to stain the population of proliferating cells in the neurogenic niches: EdU^+^ cells were fluorescently labeled with a bright photostable Alexa Fluor^®^ 488 dye in a fast, highly specific click reaction, according to the purchaser’s instruction.

Finally, slides were closed with a specific mounting (Fluoroshield, Sigma) and analyzed with a confocal microscope (Leica TCS).

### Cloning of *Notch1a* and *Notch3* and Probe Preparation

Total RNA was extracted from *N. furzeri* whole brains using miRNeasy Mini Kit (Qiagen), then total cDNA was retro transcribed from 1 μg of RNA. PCR was performed on cDNA using a GoTaq polymerase (Promega), 60°C for annealing temperature, and 60 s elongation time using the following primers:

Notch1aF: GCCCGACCATCCTTTTCTGA
Notch1aT7-R: GCAATGCAGAAGCCCTACTC-GGCCG GGACAAGTGCAATACC
Notch3F: AATGCTTGTCAAACCCGTGC
Notch3T7-R: CATCTGTCTGGATCCCTCGC-GGCCGG GACAAGTGCAATACC.
Primers were designed using the NCBI Primer-BLAST, and the RNA sequences were identified on the NCBI website and were, respectively:
*notch3* GenBank: HADY01014643.1
*notch1a* GenBank: HADY01022403.1.

0.5 μg of PCR products that contain T7 RNA polymerase promoter at the 3’ ends were used as templates for *in vitro* transcription. The sequence of the amplicon was sent for direct sequencing (Genechrome, Rome, Italy) to confirm the identity of the amplified probe.

Probes were transcribed using DIG RNA labeling kit (SP6/T7) (Roche), according to the manufacturer’s protocol.

### RNA Sequencing Data Analysis

To assess gene expression of *notch* pathway and proliferation genes, we realized graphics of RNA expression starting from RNA-seq datasets derived from publicly available and published cross-sectional experiments (Baumgart et al., 2014^1^; Kelmer et al., 2020^2^). We combined two RNA-seq datasets of brain aging in *N. furzeri*: a dataset covering five time points (5, 12, 20, 27, and 39 weeks) and five biological replicates for each age and the second containing four replicates per age (5, 12, and 39 weeks). These ages correspond to sexual maturity, young adult, adult (as defined by a decrease in growth rate), median life span, and old (∼30% survivorship) (Baumgart et al., 2014). In total, these represent 37 different samples spanning five different ages. All the pieces of information concerning the age, strain, and number of animals considered for the analysis are summarized in Table 1. We first applied the function Deseq in the suite R to normalize the counts (to have more information about the normalization algorithm used by the Deseq2 package, please refer to the paper by Love et al., 2014, and to the package manual at the link: http://www.bioconductor.org/packages/release/bioc/vignettes/DESeq2/inst/doc/DESeq2.html). We then extracted the normalized counts of the two datasets and divided them for the average expression of their respective 5 week samples to be able to combine them.

We also analyzed a publicly available proteomic dataset (Kelmer et al., 2020^3^). The dataset contains five replicates for age (5, 12, and 39 weeks, listed in Table 1) and is a combination of two separate experiments performed utilizing tandem mass tag and analyzing the same 12 week animals twice in two different contrasts: 39 vs. 12 weeks and 5 vs. 12 weeks. We therefore combined the data normalizing the protein expression dividing them for the mean value of the 12 weeks animals and plotted the resulting values. For both transcriptomics and proteomics data, we performed statistical analysis calculating the Spearman correlation value, the *p*-value adjusted, and the false discovery rate (FDR) to assess the statistical significance of the observed expression variability. All analyses were performed utilizing the suite R.

## Results

**FIGURE 1:**
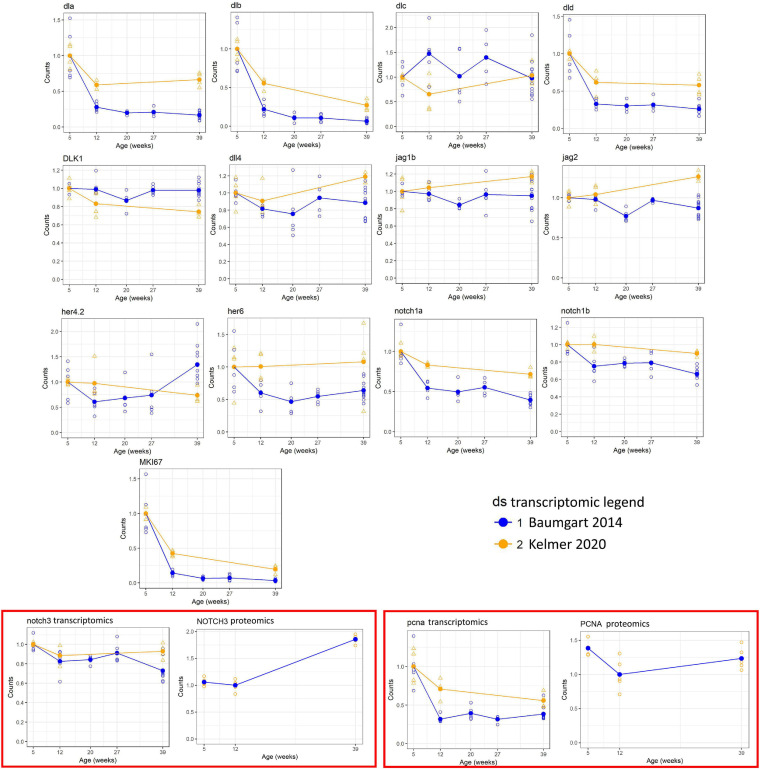
Expression of selected genes of the Notch pathway measured by RNA sequencing (RNA-seq). For each gene, the Spearman correlation coefficient, the uncorrected *p*-value, and the corrected *p*-value are shown. For correction of the *p*-value, only genes mapping to the Kyoto Encyclopedia of Genes and Genomes (KEGG) pathway dre00430 were used. Open circles represent single samples, and the solid circle represents the mean within an age step. Across-sample normalization of counts was performed using the normalization procedure of DESeq-2.

To obtain a global overview on the gene expression of members of the Notch family, we analyzed published RNA-seq (Baumgart et al., 2014), taking as the first time point of analysis the data obtained from 5-week-old animals, which corresponds to the time of sexual maturation followed by 12 weeks (young adult), 20 weeks (mature adult), 27 weeks (old), and 39 weeks (geriatric). Figure 1 reports the age-dependent regulation of gene expression coding for members of the Notch signaling pathway as identified by the Kyoto Encyclopedia of Genes and Genomes (KEGG) pathway (dre00430): eight Notch ligands, four receptors, and three effectors. Out of these, four ligands (***dla, dlb, dld***, and ***jag2***), ***notch1a***, and proliferative genes (***mki67*** and ***pcna***) showed a negative correlation with age, indicating an age-dependent downregulation of the Notch pathway, and it is consistent with an age-dependent reduction of adult neurogenesis detected previously by neuroanatomical techniques (Tozzini et al., 2012) and RNA-seq (Baumgart et al., 2016). More precisely, the observed decrease is greater in the time frame between 5 and 12 weeks of age (Figure 1), while the downregulation decelerates between 12 and 27 weeks. Statistical significance of expression changes can be assessed by the correlation and ***p***-value data listed in Table 2.

**TABLE 2:** Spearman correlation value and respective *p*-values and adjusted *p*-values for gene and proteins listed in Figure 1.

	Transcriptomics, Dataset 1 Baumgart et al., 2014			Transcriptomics, Dataset 2 Kelmer et al., 2020			Proteomics Kelmer et al., 2020		
gene	Spearman CV	pVal	FDR	Spearman CV	pVal	FDR	Spearman CV	pVal	FDR
*dla*	–7.54629E + 14	9.382986565 49819e-07	6.00511140 191884e-06	–5.91312E + 14	0.0428679750 394139	0.07620973340 34024			
*dlb*	–8.17445E + 14	1.996229181 9553e-08	3.19396669 112848e-07	–9.461E + 14	3.271765152 57932e-06	2.093929697 65076e-05			
*dlc*	–1.41441E + 13	0.447867241 867479	0.5971563224 89972	–2.95656E + 14	0.9273255929 17055	0.9572393217 20831			
*dld*	–6.64356E + 14	4.587876016 78168e-05	0.0001835150406 71267	–7.39141E + 14	0.00601894061 957786	0.0128404066 550994			
DLK1	–4.74243E + 14	0.800006284 621884	0.8258129389 64525	–7.39141E + 14	0.00601894061 957786	0.01284040665 50994			
*dll4*	–5.49124E + 14	0.769219214 254042	0.8205004952 04311	5.32181E + 14	0.07489 8436	0.1141309493 5522			
*her4.2*	4.16003E + 14	0.019930908 1685124	0.0455563615 280283	–7.39141E + 14	0.006018940619 57786	0.01284040665 50994			
*her6*	–2.25889E + 13	0.221751400 583059	0.4174144010 97524	5.91312E + 14	0.8551579930 78424	0.9121685259 50319			
*jag1b*	–5.65764E + 14	0.7624224206 48025	0.8205004952 04311	4.7305E + 14	0.1203734281 97896	0.1750886228 33304			
*jag2*	–4.05603E + 14	0.02358690140 07746	0.05031872298 83191	7.68706E + 14	0.0034824680 3771213	0.00928658143 389901			
MKI67	–8.55302E + 14	8.8838891798 5594e-10	2.84284453755 39e-08	–9.461E + 14	3.271765152 57932e-06	2.0939296976 5076e-05			
*notch1a*	–6.48132E + 14	8.064381403 0168e-05	0.000258060204 896537	–6.80009E + 14	0.0149674939 835605	0.0299349879 671209			
*notch1b*	–7.75429E + 14	3.0068523781 9565e-07	3.20730920340 869e-06	–9.461E + 14	3.2717651525 7932e-06	2.0939296976 5076e-05			
*notch3*	–5.92804E + 13	0.000441182018 497299	0.00117648538 265946	–4.13919E + 14	0.181014993 978156	0.251846948 143521	0.64253962 0399475	0.009787763993 86838	0.0261977326 229934
*pcna*	–2.04257E + 14	0.2703778006 73789	0.4806716456 42292	–8.27837E + 14	0.00088528528 9653703	0.0025753753 880835	–0.3023715913 29575	0.2733497681 76361	0.3629205847 07689

*FDR, false discovery rate.*

**FIGURE 2:**
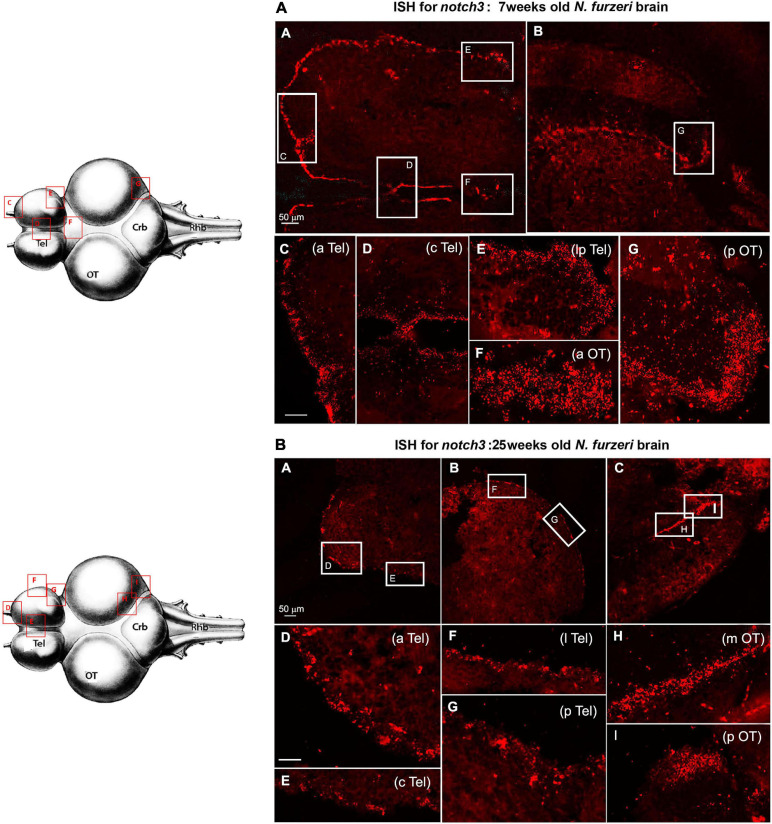
*Notch3* expression in proliferative niches of 7-weeks-old **(A**: **A–G)** vs. 25-weeks-old **(B**: **A–F)** *N. furzeri* brain**. (A) (A,B)** Overview of the brain in horizontal medial section (along the dorso/ventral axis) stained for *notch1a*, respectively, in telencephalon **(A)** and optic tectum (OT) **(B)** regions of a young *N. furzeri* brain (7 weeks). White frames show the areas magnified in panels **(C–G),** representing, respectively, the anterior (aTel), central (cTel), and lateroposterior (lpTel) telencephalic regions and the anterior (aOT) and posterior optic tectum regions. **(B) (A–C)** Overview of the brain in horizontal medial section (along the dorso/ventral axis) stained for *notch3*, respectively, in the anterior **(A)** and lateroposterior **(B)** telencephalon and the posterior optic tectum (pOT) **(C)** regions of an old *N. furzeri* brain (25 weeks). White frames show the areas magnified in panels **(D–I),** representing, respectively, the anterior (aTel), central (cTel), lateral (lTel), and posterior (pTel) telencephalic regions and the anterior (aOT) and posterior (pOT) optic tectum regions. Scale bars always represent 50 μm and are indicative for all further images without specific scale bar indication. Brain drawings on the left (modified from D’angelo, 2013–Atlas) represent the horizontal overview of the whole killifish brain; red-squared areas indicate the position of the figure panels based on the corresponding letters.

The amplitude of regulation was largest for the delta ligands (with the exception of *dlc* that, however, is ∼20 times lower expressed than the other three delta genes) and *notch1a*. Although *notch3* expression shows the same downregulation trend between 5 and 12 weeks, the overall reduction is consistently smaller, how attested by the relative RNA-seq data analysis (Figure 1) and the expression pattern in the brain tissue (Figure 2).

We were also able to find proteomic data regarding the levels of Notch3 and proliferating cell nuclear antigen (PCNA); even if the transcripts of the two genes show no evident age-dependent reduction, at the protein level, a mechanism of compensation seems to occur (Figure 1). A partial decoupling between RNA and protein level during aging already has been demonstrated in this model (Kelmer et al., 2020), and this observation could be included in this context, but from the data obtained, a Notch3 reduction can be excluded.

We decided to investigate the expression of *notch1a* (Gene ID Nfu_g_1_006683, annotated using the zebrafish ortholog ENSDARG00000052094) and *notch3* (Gene ID Nfu_g_1_007618, annotated using the zebrafish ortholog ENSDARG00000052139). As described in the *Introduction*, these genes play different roles in controlling NSC proliferation and differentiation in the zebrafish brain (Alunni et al., 2013; de Oliveira-Carlos et al., 2013). Although both genes are downregulated with age, the amplitude of regulation (i.e., 5 vs. 39 weeks) is different: 40% for *notch1a* vs. 15% for *notch3*.

In order to localize the expression domains of *notch1a* and *notch3* in the *N. furzeri* brain, we performed ISH on horizontal sections concentrating on the neurogenic niches of the telencephalon and optic tectum that we previously described in detail (Tozzini et al., 2012).

**FIGURE 3:**
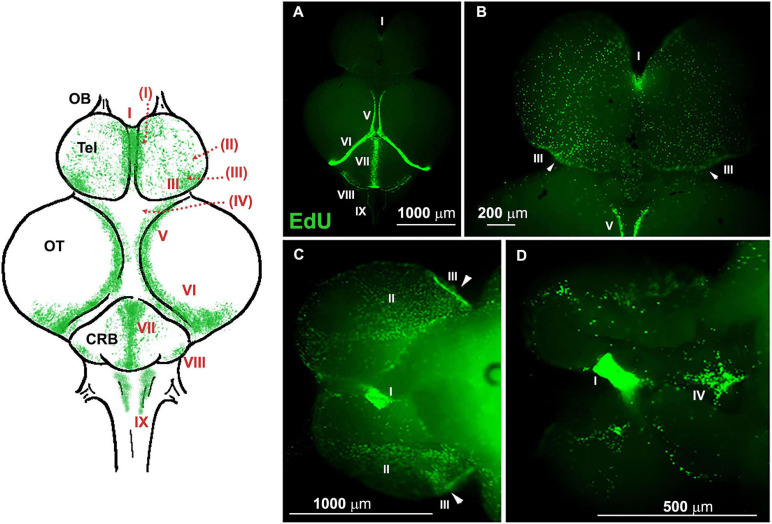
Whole-mount (WM) overview of ethynyl deoxyuridine (EdU)^+^ cells in 7-weeks-old *Nothobranchius furzeri*, 4 h after intraperitoneal injection (modified from Tozzini et al., 2012). A schematic drawing of the dorsal view of *N. furzeri* brain is shown on the *left*, where all principal brain regions are defined (OB, olfactory bulb; Tel, telencephalon; OT, optic tectum; CRB, cerebellum), and neurogenic niche distribution indicated by green dots and sequential Roman numeral indexes; dotted red lines indicate the extension of a niche (or part of it) on the ventral side of the cerebral structure, here not represented. **(A)** Dorsal view of the brain. **(B)** Telencephalic region. **(C,D)** Ventral view of the brain at lower **(C)** and higher **(D)** magnification. Nine neurogenic niches are identified in these panels: I, telencephalic niche corresponding to the subpallial region; II and III, telencephalic niches corresponding to the pallial regions; IV, preoptic niche; V, rostro-dorsal part of the niche in the optic tectum (OT); VI, caudal part of niche in the OT; VII and VIII, medial and caudal niches of the cerebellum; IX, caudal niche along the roof of the IV ventricle, visible in panel **(A)**. Arrowheads **(B,D)** indicate areas of higher concentration of proliferating cells in the caudal margin of niche III.

Figure 3 (modified from Tozzini et al., 2012) shows a whole-mount brain of young *N. furzeri*, where all neurogenic niches are visualized by incorporation of EdU and green fluorescent labeling (4 h after injection) and serves as a reference for the ensuing ISH images of the study.

**FIGURE 4:**
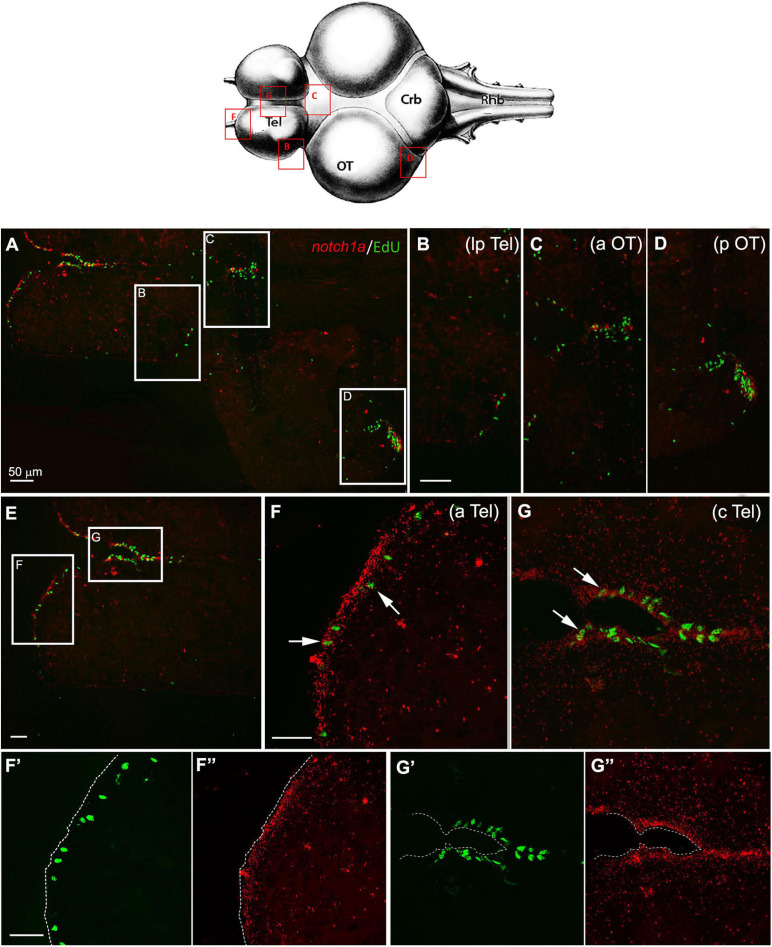
*Notch1a* expression in proliferative niches of 7-weeks-old *N. furzeri* brain. **(A)** Overview of the brain in horizontal medial section (along the dorso/ventral axis) double-stained for ethynyl deoxyuridine (EdU) in green (labeling S-phase cells) and *notch1a* in red [by *in situ* hybridization (ISH)]. White frames show the areas magnified in panels **(B–D),** representing, respectively, the lateroposterior telencephalon (lpTel), the anterior (aOT) and posterior optic tectum **(E)** representation of the telencephalic structure, labeled for EdU and *notch1*. White frames show the areas magnified in panels **(F,G),** representing, respectively, the anterior (aTel) and central (cTel) telencephalic regions. In panels **(F**’**,F**”**)** and **(G**’**,G**”**)** green and red separated channels are shown for the two regions in panels **(F)** and **(G)**. Scale bars always represent 50 μm and are indicative for all further images without specific scale bar indication. The upper brain drawing (modified from D’angelo, 2013–Atlas) represents the horizontal overview of the whole killifish brain; red-squared areas indicate the position of the figure panels based on the corresponding letters. White arrows show the presence of double-stained Edu^+^/*notch1a* cells.

The messenger of *notch1a* was coexpressed, although not exclusively limited, to active neurogenic niches, as demonstrated by double labeling with the mitotic marker EdU (Figure 4). In the telencephalon, expression of *notch1a* was mostly seen in EdU^–^ cells that were close neighbors of a *notch1a* ^–^ EdU^+^ cell, as expected, given the antimitotic activity of Notch pathway activation. A few cells were double labeled for *notch1a* and EdU (white arrows in Figures 4F,G), as already reported in zebrafish (Alunni et al., 2013). It should be noticed that *notch1a* expression in the telencephalon was more prominent in the anterior and medial parts of the telencephalon (Figures 4E–G) that correspond to a subpallial highly mitotic area (area I in Figure 3), while the number of labeled cells is smaller in the lateral margin of the telencephalon that is a pallial, low active mitotic area (area II in Figure 3). Expression becomes higher in a lateroposterior area where mitotic activity is moderate (Figure 4B, area III in Figure 3). In the optic tectum, expression of *notch1a* is strictly limited to neuroepithelial stem cell niches (Figures 4C,D) where partial double labeling of *notch1a* and EdU is observed.

**FIGURE 5:**
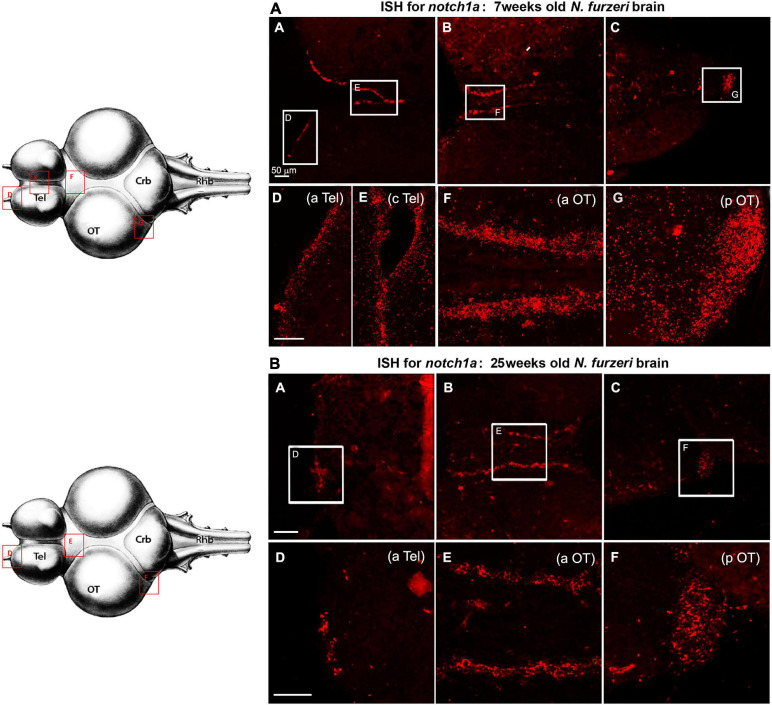
*Notch1* expression in proliferative niches of 7-weeks-old **(A**: **A–G)** vs. 25-weeks-old **(B**: **A–F)** *N. furzeri* brain. **(A) (A–C)** Overview of the brain in horizontal medial section (along the dorso/ventral axis) stained for *notch1a*, respectively, in telencephalon **(A)**, anterior optic tectum (aOT) **(B),** and posterior optic tectum (pOT) **(C)** regions of a young *N. furzeri* brain (7 weeks). White frames show the areas magnified in panels **(D–G),** representing, respectively, the anterior (aTel) and central (cTel) telencephalic regions and the anterior (aOT) and posterior optic tectum regions. **(B) (A–C)** Overview of the brain in horizontal medial section (along the dorso/ventral axis) stained for *notch1a*, respectively, in telencephalon **(A)**, anterior optic tectum (aOT) **(B)** and posterior optic tectum (pOT) **(C)** regions of an old *N. furzeri brain* (25 weeks). White frames show the areas magnified in panels **(D–G),** representing, respectively, the anterior (aTel) and central (cTel) telencephalic regions and the anterior (aOT) and posterior (pOT) optic tectum regions. Scale bars always represent 50 μm and are indicative for all further images without specific scale bar indication. Brain drawing on the left (modified from D’angelo, 2013–Atlas) represents the horizontal overview of the whole killifish brain; red-squared areas indicate the position of the figure panels based on the corresponding letters.

In agreement with the RNA-seq data (Figure 1), expression of *notch1a* markedly declines during aging in all neurogenic niches without changing the regional pattern of labeling (Figure 5).

**FIGURE 6:**
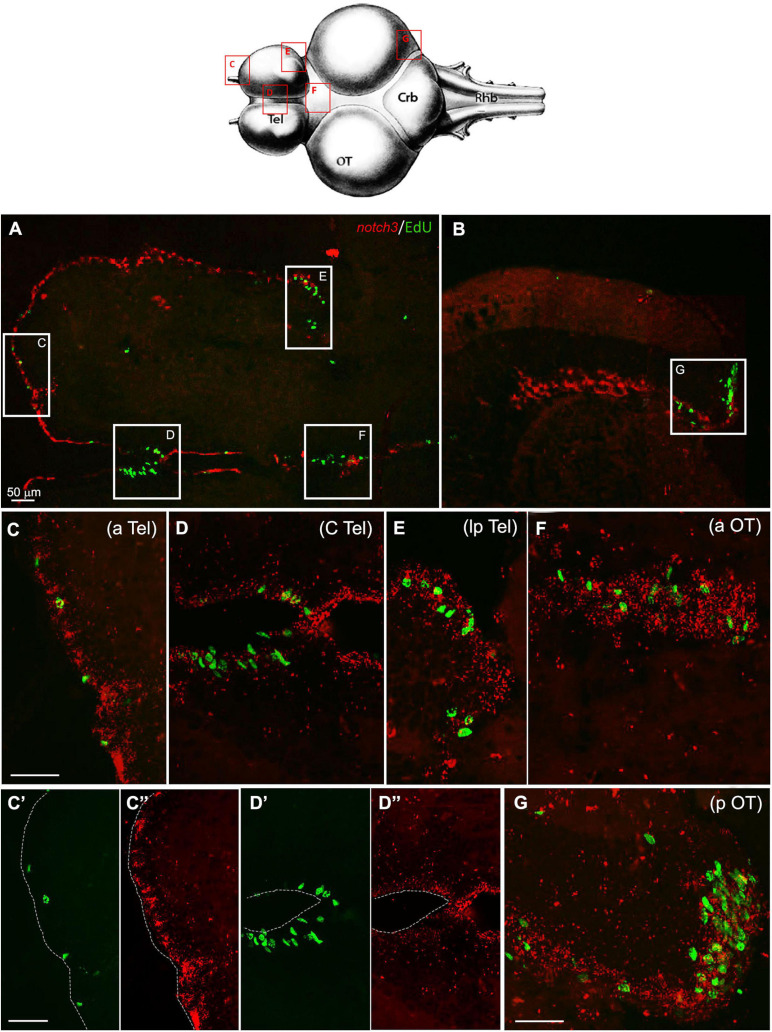
*Notch3* expression in proliferative niches of 7 weeks-old *N. furzeri* brain: **(A)** Overview of the brain in horizontal medial section (along the dorso/ventral axis) double-stained for EdU in green (labelling S-phase cells) and *notch3* in red (by ISH). White frames show the areas magnified in **(C–G)**, representing respectively the anterior (aTel), central (cTel), latero-posterior Telencephalon (lpTel), the anterior (aOT) and posterior (pOT) Optic Tectum regions - In **(C’)/(C”)** and **(D’)/(D”)** green and red separated channel are shown for the two regions in **(C,D)** - Scale bars always represent 50μm and are indicative for all further images without specific scale bar indication. Footprint (modified from D’angelo, 2013–Atlas) represents the horizontal overview of the whole killifish brain; red-squared areas indicate the position of the figure panels, based on the corresponding letters. White arrows show the presence of double-stained Edu^+^/*notch3* cells.

Although the RNA-seq data show a minor drop in the *notch3* expression between 5 and 12 weeks of age, our ISH results suggest that already at 7 weeks, we can observe a level of *notch3* RNA expression that is comparable to the level observed at 27 weeks of age. As opposed to *notch1a*, the expression of *notch3* was not limited to active neurogenic niches (EdU positive), but it was equally observed in all stem cell niches, regardless of the visible mitotic activity level in the region (Figure 5). In the young adult telencephalon, expression of *notch 3* was similar in regions that vary greatly in mitotic level: the subpallial highly mitotic area (Figures 6A–D, area I in Figure 3) throughout the frontal margin of the telencephalon, that is, a pallial, lower active mitotic area (Figures 6A–C), and in the lateroposterior area with moderate mitotic activity (Figures 6A–E, area II and III in Figure 3). In the optic tectum, expression of *notch3* is not limited to neuroepithelial stem cell niches (Figures 6F–G, anterior and posterior margin niches of the OT, respectively), but extends into the ventricular margin of the optic tectum (Figure 6B) which clearly shows only a red staining and it is formed by radial glia with extremely low mitotic activity (Tozzini et al., 2012).

## Discussion

In conclusion, by comparing the RNA-seq results with what has emerged from the histological analysis of the ISH experiments, both expression pattern and regulation differ between *notch1a* and *notch3*. Expression of the former is limited to mitotically active regions and consequently is greatly reduced by aging. Expression of the latter is observed in all cells that have a neurogenic potential, regardless of the level of their actual mitotic activity, and is less influenced by age both at the transcript and protein levels.

The reduction of the expression of *notch1a* (as well as that of *mki67* and *pcna*) shows the bigger amplitude between the ages of 5 and 12 weeks, the period in which the brain grows considerably. Based on the techniques utilized in this work (RNA-seq data analysis and ISH), it cannot be formally excluded that the reduction observed could be due to the relative reduction in the fraction of cells expressing *notch1a* on the total amount of cells in the brain. However, the fact that its normalized expression is reduced at the brain level is nonetheless a significant observation. Typically, all pathways involved in growth and differentiation are strongly involved in the metabolic processes that determine the future aging and life span of an individual; it also has been demonstrated that the specific expression of metabolic-related genes already at early life stages could be predictive of life expectancy of *Nothobranchius* (Baumgart et al., 2016). Thus, growth and aging are two processes that are strictly correlated and cannot be easily disentangled.

On the other hand, *notch3* transcript seems to be less affected by aging and even to increase its protein level over time. Unfortunately, the dataset that we analyzed did not contain information about Notch1a or other Notch proteins.

From the literature, it is known that the reduction of *notch1a* induces stem cells to divide asymmetrically, whereas the reduction of *notch3* promotes symmetrical divisions (Alunni et al., 2013; Than-Trong et al., 2018). Thus, a different antiproliferative role could be inferred for the two genes, depending on the differential proliferation outcome.

Our results show that in *N. furzeri* telencephalon, *notch1a* is expressed, although not exclusively, in proliferating regions, mainly localizing in close proximity to EdU^+^ cells, areas in which the expression of *notch1a* seems to be more concentrated.

In zebrafish, it has been described that the high majority of proliferative radial glia and about 50% of proliferative non-radial glia express *notch1a* (de Oliveira-Carlos et al., 2013), while in killifish, we observed that almost all proliferative Edu^+^ cells of the telencephalic niches do not express *notch1a*, supporting the notion of the antiproliferative role of this gene, as also supported by the work of Chapouton et al. (2011), in which they showed the mutually exclusive expression of proliferative markers and effectors of Notch pathway. The limited number of double-stained EdU-*notch1a* cells (Figure 4 white arrows) could represent cells lacking the expression of *notch3*, thus undergoing the symmetrical above the asymmetrical division (inhibited by *notch1a* expression). These cells could belong to the pool of slow-cycling, glial-like NSCs, already observed in our previous work where double-stained S100^+^/EdU^+^ cells were present in this area in exiguous number (Tozzini et al., 2012).

In zebrafish telencephalon, *notch3* has been described to have an expression pattern similar to *notch1a* in radial glial cells but to be rarely expressed in the non-glial proliferative population (de Oliveira-Carlos et al., 2013). In our observations, *notch3* shows a more widespread area of staining with respect to the neurogenic niches, but also in this case, we could identify a few double-labeled EdU^+^/*notch3*^+^ cells.

In zebrafish optic tectum, the majority of proliferating cells are reported to express *notch1a* and *notch3* (de Oliveira-Carlos et al., 2013). In our case, *notch1a* expression resembles closely the situation already described for the telencephalon: very few cells, both in the anterior and posterior neurogenic niches, present a double Edu/*notch1a* staining. On the contrary, *notch3* seems to have a higher level of colocalization with EdU^+^ cells with respect to *notch1a.* The interpretation of this finding is ambiguous: it is known that neurogenic niches of the optic tectum have in fish a neuroepithelial origin (Ito et al., 2010) and are characterized by fast-cycling cells supposedly undergoing symmetrical divisions. It is possible that in these areas, *notch3* does not have the canonical function of symmetrical division inhibition. To assess that, further investigations would be needed.

Taken together, our observations finally suggest that, as already demonstrated for zebrafish (Alunni et al., 2013), also in our model, a possible differential role of the two receptors could exist in neurogenic activity control by acting on distinct cellular subpopulations, so to realize a fine-tuned regulation of the proliferative potential of the niches throughout the entire fish life.

## Data Availability Statement

Publicly available datasets were analyzed in this study. This data can be found here: Baumgart et al. (2014): http://www.ncbi.nlm.nih.gov/geo/query/acc.cgi?acc=GSE52462; Kelmer et al. (2020): http://www.ncbi.nlm.nih.gov/geo/query/acc.cgi?acc=GSE125373; Kelmer et al. (2020): http://www.ebi.ac.uk/pride/archive/projects/PXD012314.

## Ethics Statement

The animal study was reviewed and approved by Italian Ministry of Health (Number 96/2003a).

## Author Contributions

ETT designed the study. ETT and SB performed *in situ* hybridization, confocal acquisition, imaging, and wrote the manuscript. SB performed RNAseq analysis. Both authors read and approved the final manuscript.

## Conflict of Interest

The authors declare that the research was conducted in the absence of any commercial or financial relationships that could be construed as a potential conflict of interest. The reviewer, CL, declared a past collaboration with one of the authors, ETT, to the handling editor.

## Footnotes

1 http://www.ncbi.nlm.nih.gov/geo/query/acc.cgi?acc=GSE52462

2 http://www.ncbi.nlm.nih.gov/geo/query/acc.cgi?acc=GSE125373

3 http://www.ebi.ac.uk/pride/archive/projects/PXD012314
